# Recent Developments in Ultrafine Shape Memory Alloys Using Amorphous Precursors

**DOI:** 10.3390/ma16237327

**Published:** 2023-11-24

**Authors:** Sung-Hwan Hong, Hae-Jin Park, Gi-An Song, Ki-Buem Kim

**Affiliations:** 1 Department of Nanotechnology and Advanced Materials Engineering, Sejong University, Seoul 05006, Republic of Korea; haejinp@sejong.ac.kr; 2 Division of Advanced Materials Engineering, Kongju National University, Cheonan-si 31080, Republic of Korea; gasong@kongju.ac.kr

**Keywords:** amorphous precursor, shape memory alloy, crystallization kinetics, shape memory effect, superelasticity

## Abstract

In this review, we systematically reviewed the recent advances in the development of ultrafine shape memory alloys with unique shape memory effects and superelastic behavior using amorphous metallic materials. Its scientific contribution involves defining and expanding the range of fabrication methods for single-phase ultrafine/nanocrystalline alloys with multicomponent systems. In multicomponent amorphous alloys, the crystallization mechanism depends on the alloy composition and is a selectable factor in the alloy designing method, considering the thermodynamic and physical parameters of constituent elements. The crystallization kinetics can be controlled by modulating the annealing condition in a supercooled liquid state with consideration of the crystalline temperature of the amorphous alloys. The phase stability of austenite and martensite phases in ultrafine shape memory alloys developed from amorphous precursors is determined according to alloy composition and grain size, which strongly influence the shape memory effect and superelastic behavior. A methodological framework is subsequently suggested to develop the ultrafine shape memory alloys based on the systematic alloy designing method, which can be considered an important strategy for developing novel ultrafine/nanocrystalline shape memory alloys with excellent shape memory and superelastic effects.

## 1. Introduction

NiTi shape memory alloys (SMAs) have been a subject of interest due to the remarkable attractiveness of their shape memory effect (SME) and superelastic effect (SE) [1,2]. Based on these unique characteristics, NiTi SMAs have been widely applied as smart materials in automotive, aerospace, and biomedical applications [3,4,5,6]. The SME and SE effects originate from the strain-recoverable martensitic transformation between the B2 austenite phase and the B19’ martensite phase with change a in temperature and stress conditions [7,8]. Therefore, the martensitic transformation behavior of SMAs, depending on the transformation temperatures, transformation path, and thermal and stress hysteresis, determines the transformation strain, operating temperature, cycling stability, and fatigue life during use in applications. The binary NiTi SMAs known as “Nitinol” [5] have intrinsic drawbacks, such as a limited martensitic transformation temperature below 373 K, relatively low strength (less than 700 MPa), and fatigue degradation during stress and thermal cycling [1,9,10,11,12,13]. To tailor these restricted characteristics of NiTi SMAs, alloy designing and thermomechanical processing, such as cold-rolling, forging, drawing, high-pressure torsion (HPT), equal channel angular pressing (ECAP), and surface mechanical attrition treatment (SMAT), have been widely studied [10,14,15,16,17].

As a result of intensive studies on alloy design, the SME and SE of NiTi-based SMAs have been significantly improved through a multicomponent alloying strategy, with the addition of solute elements that are able to dissolve into the NiTi intermetallic compound by substituting Ni or Ti. To increase the transformation temperature of SMAs, ternary alloy systems have been developed with the addition of ternary alloying elements. The addition of Cu and Mn has relatively little effect on the transformation temperatures of NiTi SMAs and leads to a change in the transformation path from B2–B19’ to B2–B19–B19’, which appears due to the relative phase stability between the three competing martensite phases (B19’, B19, and R phases) with respect to the B2 austenite phase [18]. The addition of other alloying elements, such as Hf, Au, Pd, and Pt, in NiTi-based SMAs dramatically increases the transformation temperatures above 423 K proportional to the number of additional elements [19,20]. These alloying elements in ternary alloy systems have important roles to play in terms of modifying the transformation temperatures, transformation behavior, and crystal structure of the martensite phase [15,16]. Nevertheless, the relatively easy plastic deformation of the ternary NiTi-based SMAs due to their low yield strength at room temperature is still considered a challenge [21,22,23]. In order to improve the strength of the NiTi-based SMAs, quaternary alloy systems have been discovered based on a ternary Ni–Ti–Hf system, presenting highly improved strength, transformation stress, transformation strain, and damping capacity [24,25,26,27,28]. Karaca et al. [26] reported that the addition of Pd to a ternary Ni–Ti–Hf SMA resulted in highly improved yield stress levels (~2 GPa) in the alloys with a high damping capacity of 35 J/cm^3^. The Ni–Ti–Hf–Zr SMAs reported by Hong et al. [29] exhibited highly elevated transformation temperatures (up to 830 K) with a high yield strength of ~1.6 GPa at room temperature. These improvements in Ni–Ti–Hf–Zr SMAs originate from the severe lattice distortion during martensitic transformation with an increase in Zr content. However, quaternary NiTi-based SMAs usually exhibit brittle behavior under compression due to their limited plasticity [28,29]. The poor workability of SMAs has been considered one of the major problems for thermomechanical processing to further improve their mechanical properties, SME, and SE by tailoring the microstructural features of the alloys.

Grain refinement via severe plastic deformation (SPD) is an effective method to enhance the strength, strain recovery behavior, and fatigue life of SMAs [30,31,32,33,34]. For nanocrystalline NiTI SMAs fabricated via SPD, an excellent combination of static functional properties, high recovery stress, and completely recoverable strain has been achieved. In addition, the SMAT process is applied to NiTi SMAs to produce the grain size gradient (GSG) from the surface to the inside [14]. The GSG surface layers with grain sizes of 5–100 nm result in very high hardness values of 5.7–4.5 GPa and compressive residual stresses up to 1093 MPa, which notably suppress crack nucleation. The SPD method for the grain refinement of the alloys has been also investigated for ternary Ni–Ti–Hf SMAs [35,36,37]. Kockar et al. [35] reported that equal channel angular extrusion (ECAE) of Ni–Ti–Hf SMAs increases the critical stress for slips via grain refinement, which improves their thermocycle stability and transformation strain. Shuitcev et al. [37] found that the HPT process is much more effective in the refinement of the microstructure of Ni–Ti–Hf SMAs.

In recent decades, nanocrystalline SMAs with enhanced physical characteristics have been developed. One of the strategies to develop ultrafine NiTi-based SMAs involves using multicomponent amorphous alloy precursors (AAPs). The amorphous alloys (AAs) exhibit a glass transition into a meta-stable supercooled liquid state between the glass transition temperature (T_g_) and the crystallization temperature (T_x_), which provides an opportunity to use them as a precursor for the development of ultrafine-structured alloys [38,39,40]. The crystallization mechanism of AAs is determined by their alloy composition. Therefore, suitable AA compositions with polymorphous crystallization mechanisms considering phase transformation into single-phase SMAs have been investigated for NiTi-based multicomponent alloy systems [41,42]. The crystallization kinetics of AAPs have been investigated to achieve nano-/ultrafine-scale single-phase SMAs.

In the above context, in Section 2, we review the mechanism of nanocrystallization in NiTi-based SMAs via thermomechanical processing and the influence of grain size on physical and thermoelastic characteristics. Section 3 summarizes the recent progress in the development of ultrafine SMAs fabricated using multicomponent AAPs. The influence of alloying elements and heat treatment conditions on the crystallization kinetics, nanostructure, thermal stability, and mechanical properties of SMAs is also discussed.

## 2. Nanocrystalline NiTi-Based Shape Memory Alloys Fabricated Using Thermomechanical Processing

### 2.1. The Effect of Grain Size on the Superelastic Effect of Binary NiTi Shape Memory Alloys

The microstructure of NiTi SMAs and their microscopic properties such as SME and SE have a close relationship. Recently, great efforts have been made in the investigation of the thermomechanical properties of nanocrystalline NiTi SMAs fabricated by SPD processing to reduce their grain size to nanoscale. The major difference between the nanocrystalline NiTi SMAs and the coarse-grained SMAs is the high density of the grain boundary [43,44], leading to grain refinement strengthening [37], a linearized stress–strain relation with vanishing hysteresis, a broadened superelasticity window [45], and improved cyclic and dynamic stability [32,46,47,48].

**Figure 1:**
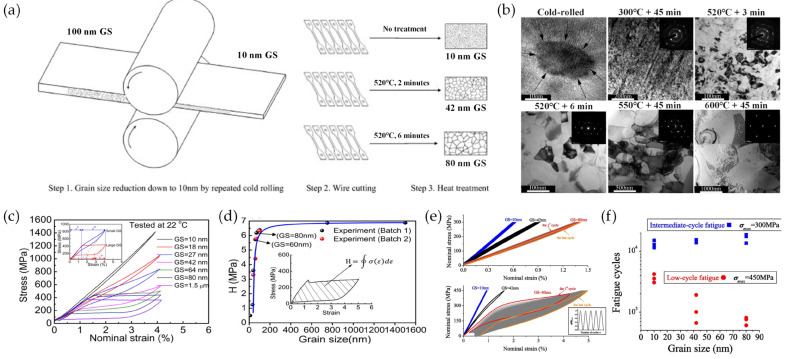
(**a**) Schematic drawing of the cold-rolling and heat treatment processing of a NiTi SMA plate, (**b**) transmission electron microscopy (TEM) bright-field (BF) images, (**c**) stress–strain curves, (**d**) variation in the measured hysteresis loop area (H), (**e**) stress–strain responses during the stress-controlled fatigue failure test, (**f**) fatigue lives under two different controlled stress levels of the as-rolled and heat-treated alloys [32,45].

As shown in Figure 1a, nanocrystalline NiTi SMA plates can be produced using cold rolling to reduce the grain size to 10 nm [32]. After cold-rolling processing, the grain size of as-rolled NiTi SMAs is tailored in the nanoscale (10–80 nm) using heat treatment processing and strongly depends on the heat treatment temperature and times (Figure 1b) [45]. Heat treatment at 300 °C promotes partial devitrification and recovery of the deformed lattice, and a higher heat treatment temperature produces a fully recrystallized microstructure with grain growth. With the decrease in grain size, the phase transformation stress increases and the hysteresis loop area (*H*) decreases monotonically (Figure 1c). For the total strain, the elastic strain increases and the portion of transformation strain decreases due to a gradual suppression of stress-induced phase transformation with the grain size reduction, which, at the same time, promotes elastic strain. Therefore, the grain size reduction in nanocrystalline NiTi SMAs modifies the isothermal *H* during loading−unloading cycles. When the grain size is larger than 80 nm, there is little change in the *H*, whereas when the grain size is smaller than 60 nm, the *H* decreases rapidly (Figure 1d). This means that nanocrystalline NiTi SMAs with extremely small grain sizes behave very differently from the conventional coarse-grained NiTi SMAs with grain sizes larger than 100 nm in terms of their macro-mechanical properties. The strong grain size dependence of *H* on loading−unloading cycles is strongly correlated with the internal length scale, i.e., the grain size and austenite/martensite interface thickness, of the alloys in the energetics and dynamics of the phase transformation. The isothermal stress hysteresis means mechanical energy dissipation during a phase transition cycle. As the phase transition proceeds, the alloys experience many instabilities via nucleation and growth of martensite domains and energy is dissipated, which is equal to the free energy change [49]. For the stress-induced phase transformation, hysteresis originates from the nucleation of martensite in individual grains as a result of numerous microscopic instabilities in the austenite phase. When the grain size is reduced below a critical size, the specific interfacial energy term overwhelmingly dominates the total energetics of the alloys. As such, the alloys become more stable and hysteresis tends to vanish [50,51].

Yin et al. [32] investigated the influence of grain size on the fatigue life of superelastic NiTi SMAs during repetitive loading−unloading cycling. As shown in Figure 1e, the cyclic stress−strain responses of SMAs with different grain sizes were mainly in the elastic region, with little phase transformation under low-stress cyclic loading of 300 MPa. The strain amplitude reduced with a decrease in grain size ranging from 1.4% to 0.6%. Under higher stress cyclic loading of 400 MPa, the strain amplitude was 4.5% for the alloy with a grain size of 80 nm and a residual strain of 0.64%, while the alloys with lower grain sizes (42 nm and 10 nm) exhibited low strain amplitude in the elastic region with little phase transformation due to the higher elastic modulus. The fatigue lives of the NiTi SMAs with different grain sizes on the nanoscale are shown in Figure 1f. For the low-stress cyclic loading condition, the alloys exhibited a similar order of magnitude, with an average fatigue life of 15,219 cycles, while the fatigue life of alloys under the high-stress cyclic loading condition significantly increased with grain size reductions. When the strain amplitude is small, the grain size has little effect on the fatigue life due to deformation occurring in the elastic region. However, when phase transformation involves deformation, the grain size significantly influences the fatigue life of nanocrystalline NiTi SMAs. The enhancement in fatigue life is mainly attributed to the effects of grain size reduction on both strengthening and hysteresis reduction during the cyclic phase transformation. For conventional NiTi SMAs with coarse grains, the *H* strongly correlates with the fatigue life of the material [52]. On the other hand, such correlation works break down in high cycle fatigue in nanocrystalline NiTi SMAs. There are two microstructural factors responsible for the strong grain size effect [1,45,50,53,54]. The first factor is the rapid increase in the volume fraction of the grain boundary, which acts as a non-transformable layer. When the grain size is reduced to the nanoscale, the mechanical constraint of the grain boundary against the deformation of crystal becomes very strong compared to coarse grains. The second factor is the rapid increase in the volume fraction of the phase boundary, which acts as a transition layer separating the lattice of the austenite and martensite phases. Inside the coarse grains, the phase boundary is generated in the nucleation process under stress and can move reversibly with the reversal of external driving. In the nanograins, however, energetic and dynamic features of the phase boundary change due to the rapid increase in its volume fraction, which results in strengthening with grain size reduction and a decrease in the *H* of a superelastic cycle.

**Figure 2:**
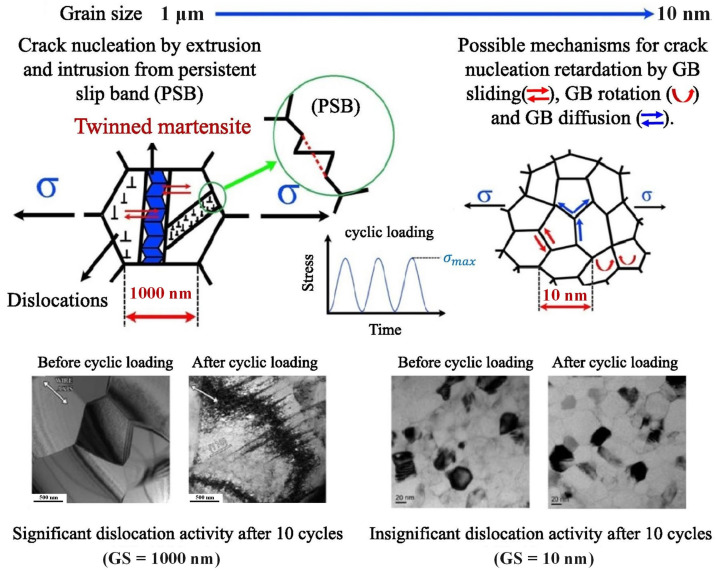
The effects of grain size on the possible change from the dislocation-dominated crack nucleation mechanism at the micro-scale to the grain boundary-mediated mechanism at the nanoscale, where the white arrows indicate the wire axis [32,60].

Regarding the possible fatigue mechanism, the role of grain size in the change in the fatigue mechanism from dislocation-dominated crack nucleation at the micro-scale to the grain boundary-mediated mechanism at the nanoscale has been suggested (Figure 2) [32,55,56,57,58,59,60]. The fatigue of SMAs involves both a phase transition that is reversible at the lattice level and the generation of a defect that is irreversible and permanent, such as dislocation and deformation twins in austenite and martensite [61,62,63,64]. Additionally, it is key that the reversible phase boundary motion is accompanied by the generation of dislocation structures and damage accumulation inside a grain under external cyclic stress or strain. The phase transformation itself is realized by distinct, successive domain nucleation and growth events of a first-order nature inside the coarse grain. In the coarse-grained (~1000 nm) alloys, dislocations were indeed created during the mechanical cycling, and the density of dislocation defects significantly increased. On the other hand, no clear evidence for dislocation activity could be found in the TEM investigation of nano-grained (~10 nm) alloys [60]. The suggested possible mechanism for crack nucleation in nanocrystalline NiTi SMAs is that grain boundary sliding, grain boundary rotation, and grain boundary diffusion are favored during cyclic loading due to the high resistance against dislocation slip upon cycling.

Understanding the microstructural evolution and the related mechanical properties of binary NiTi SMAs, it is concluded that the grain size of binary NiTi SMAs can be refined to the nanoscale using the SPD and heat treatment methods, which strongly influence the phase transformation and related mechanical response. With the decrease in grain size, stress-induced martensitic transformation is suppressed gradually, which decreases the transformation strain and *H* during cyclic loading. The *H* significantly depends on the grain size due to the increase in the grain boundary density and the austenite/martensite interface thickness, which suggests that the specific interfacial energy term overwhelmingly dominates the total energetics of the alloy. Therefore, the *H* of a superelastic cycle tends to vanish. Moreover, the fatigue mechanism of nanocrystalline SMA is changed to a grain boundary-mediated mechanism due to the high resistance against dislocation slip.

### 2.2. Phase Transformations in Nanocrystalline NiTi Shape Memory Alloys

Martensitic transformations between austenite and martensite can be induced by the application of stress and temperature due to the fact that differences in elastic energy caused by the change in the modulus of elasticity serve as a driving force for the transformation, similar to the effect of the lattice distortion of the martensite on the transformation. Therefore, if the transformation involves a large change in the modulus of elasticity, the relationship between the critical stress and the temperature for a thermoelastic martensitic transformation is non-linear [65]. In the nanocrystalline NiTi SMAs, the martensitic transformation is suppressed with decreasing grain size [44,45]. This implies that the transformed volume fraction decreases with the decreasing grain size, and the onset of the martensitic transformation is shifted toward lower temperatures. Therefore, the martensite start temperature (M_s_) drops below the transformation temperature of the R-phase in grains smaller than about 150 nm, leading to a two-step transformation from B2 austenite via the R-phase to B19’ martensite. Moreover, the martensitic phase transformation is suppressed in grains smaller than 60 nm under cyclic stress or temperature due to the introduction of elastic strains and crystal refinement [60,66]. In coarse-grained NiTi alloys, martensite nucleates in dislocation tangles in the matrix and near grain boundary dislocations [44], whereas, in the case of nanocrystalline NiTi alloys, the grain boundaries act as heterogeneous nucleation sites, indicating that grain boundaries can favor martensitic transformation [67]. In grains smaller than 60 nm, there is no dislocation and little elastic strain in the grains [60]. Therefore, it is concluded that the martensitic transformation is suppressed due to an increased energy barrier caused by high elastic strain energy originating from the high volume fraction of grain boundaries [44].

**Figure 3:**
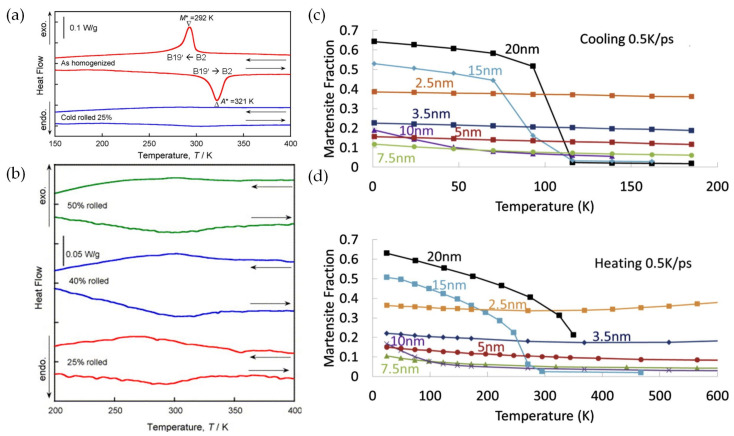
DSC cycling curves of (**a**) as-homogenized coarse-grained and 25% cold-rolled NiTi SMAs, (where M* and A* are peak temperatures for martensitic- and austenitic-transformation, respectively) and (**b**) aged nanocrystalline NiTi SMA at 573 K for 3.6 ks after 25%, 40%, and 50% cold-rolling. The martensite fraction as a function of temperature during (**c**) cooling and (**d**) heating in a nanocrystalline NiTi SMA with average grain size of 10 nm [68,69].

Tsuchiya et al. [68] experimentally measured the phase transformation temperature of nanostructured NiTi SMAs using differential scanning calorimetry (DSC). The coarse-grained NiTi SMAs exhibit obvious martensitic phase transformation between the B19’ martensite phase and the B2 austenite phase, with peak temperatures of 321 K and 292 K under continuous heating and cooling conditions (Figure 3a), whereas the nanocrystalline NiTi SMAs with grain size of 5–37 nm fabricated via heat treatment after cold rolling processing exhibit no evidence for martensitic phase transformation during heating and cooling in a temperature range between 200 K and 400 K. Morrison et al. [69] also reported a temperature-induced martensitic transformation in nanocrystalline SMA with realistic grains using a first molecular dynamics (MD) simulation. The martensite fraction as a function of temperature during cooling and heating for various grain sizes is shown in Figure 3c,d. The nano-grained alloys with grain sizes of 15 and 20 nm undergo clear martensitic transformations. Reducing the grain size reduces the transformation temperatures and induces a more gradual transformation. For the below-critical grain size of 10 nm, the transformation is completely hindered because the mechanical constraints imposed by neighboring grains with different orientations hinder nucleation in grain boundaries.

For the phase transformation behavior of nanocrystalline SMAs, it is concluded that martensitic transformation is significantly shifted toward lower temperatures with decreasing grain size, and the transformation path can be changed from one step (B2 to B19’) to two steps (B2 to R to B19’) if the grain size is smaller than the critical size. Moreover, the highly dense grain boundaries induce high elastic strain energy, resulting in an energy barrier for phase transformation, which suppresses martensitic transformation during cyclic stress and temperature.

### 2.3. Nanocrystalline Ternary NiTi-Based Shape Memory Alloys

**Figure 4:**
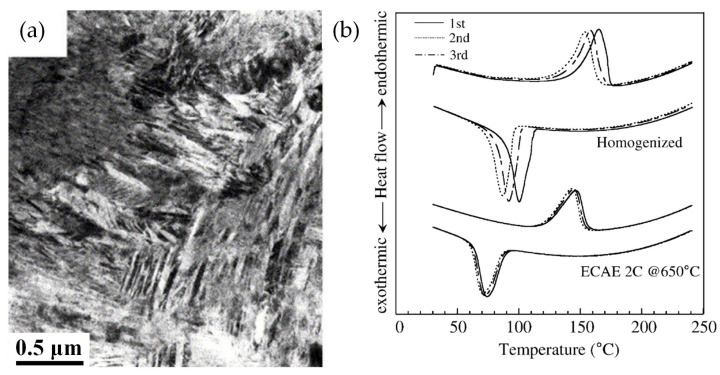
(**a**) TEM BF image of Ni_49.8_Ti_42.2_Hf_8_ SMA that was ECAE-processed at 650 °C, (**b**) DSC cyclic curves of the homogenized and ECAE-processed Ni_49.8_Ti_42.2_Hf_8_ SMAs [35].

For the nanocrystalline binary NiTi SMAs, the martensitic transformation temperature is shifted toward lower temperatures depending on the grain size [44,45,66,68,69]. On the other hand, the nanocrystalline ternary NiTi-based SMAs have different martensitic transformation behavior in comparison with nanocrystalline binary NiTi SMAs. In coarse-grained NiTi-based SMAs, the addition of alloying elements through substitution with Ni or Ti significantly increases the martensitic phase transformation temperatures [18,19,20,28,29]. Particularly, the addition of Hf to NiTi SMAs rapidly increases the peak martensite temperature from 342 K to 798 K [70]. Therefore, NiTiHf SMAs with high transformation temperatures have attracted significant attention from engineering fields. To improve the mechanical properties, thermomechanical responses, and cycle stability of the ternary Ni–Ti–Hf SMAs, grain refinement by SPD has been conducted [35,36,37]. Kockar et al. [35] reported the influence of grain size on the cyclic reversibility of Ni_49.8_Ti_42.2_Hf_8_ SMA. By conducting ECAE processing at 650 °C for the homogenized alloy, nano-grained NiTiHf SMAs consisting of the martensite phase with grain sizes of 300–500 nm were successfully fabricated (Figure 4a). The phase transformation temperatures of the homogenized alloys with a grain size of 45 μm decreased during thermal cycling, whereas the nano-grained NiTiHf SMAs demonstrated a significant improvement in the thermal stability of phase transformation temperatures compared to the homogenized alloy (Figure 4b).

**Figure 5:**
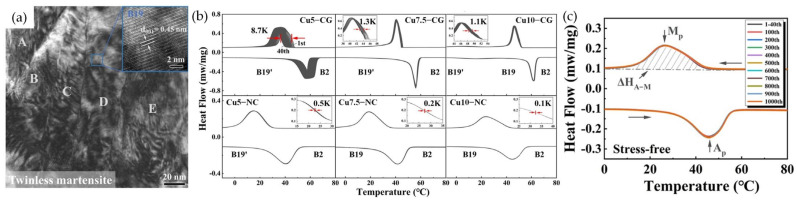
(**a**) TEM BF image of nanocrystalline Ni_40_Ti_50_Cu_10_ SMA, where capital letters indicate twiness martensite plates, (**b**) DSC cycling curves of coarse-grained and nano-grained Ni–Ti–Cu SMAs with different Cu contents, (**c**) DSC cycling curves of nanocrystalline Ni_40_Ti_50_Cu_10_ SMA for 1000 thermal cycles [71].

Further advances in the thermal stability of transformation during thermal cycling of ternary NiTi-based SMAs were achieved by developing nanocrystalline Ni–Ti–Cu SMAs [71]. The addition of Cu into NiTi SMAs induced an improvement in lattice compatibility, which reduced the generation of defects during phase transformation [72]. The nano-grained Ni–Ti–Cu SMAs produced via cold rolling with a thickness reduction ratio of 40% followed by annealing at 400 °C for 0.5 h exhibited twinless martensite plates with a size of 50 nm (Figure 5a). Comparing coarse and nano-grained Ni–Ti–Cu SMAs (Figure 5b), the shift in transformation temperatures during thermal cycles was reduced with an increase in Cu content in both the coarse and nano-grained alloys, where the nano-grained alloys exhibited smaller changes in transformation temperatures. Therefore, it was concluded that the addition of Cu and the nano-grained structure improved the thermal cyclic stability of phase transformation temperatures. As shown in Figure 5c, nanocrystalline Ni–Ti–Cu SMA also revealed the excellent reversibility of the stress-free temperature-induced martensitic transformation during 1000 thermal cycles.

**Figure 6:**
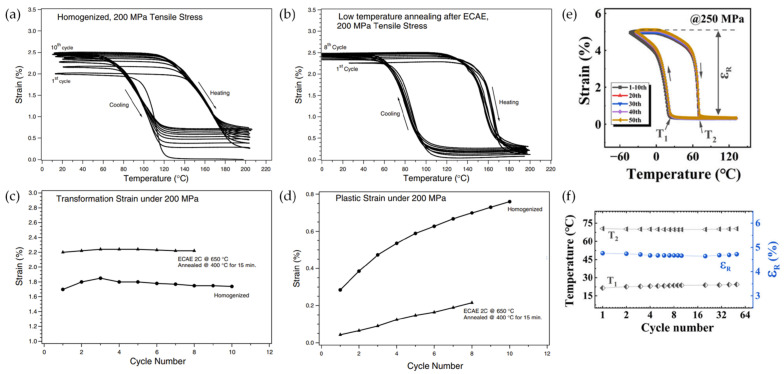
(**a**,**b**) Cyclic strain–temperature curves under 200 MPa, (**c**) transformation strain, and (**d**) accumulated plastic strain in coarse-grained (homogenized) and nano-grained (annealed after ECAE processing) Ni_49.8_Ti_42.2_Hf_8_ SMAs; (**e**) cyclic strain–temperature curves under 250 MPa and (**f**) transformation strain in nano-grained Ni_40_Ti_50_Cu_10_ SMAs [35,71].

For the thermomechanical responses, the nanocrystalline ternary Ni–Ti–Hf and Ni–Ti–Cu SMAs showed excellent cyclic stability and large recovery strain under constant tensile stress. Kockar et al. reported [35] that the homogenized Ni–Ti–Hf SMA with coarse grains exhibited a transformation strain of ~1.7% with irrecoverable plastic deformation (~0.76%) during thermal cycling under 200 MPa (Figure 6a,c,d). On the other hand, the nano-grained Ni–Ti–Hf SMA fabricated through annealing at 400 °C after ECAE processing exhibited an improved transformation strain of ~2.5% with a lower plastic strain (~0.1%) during thermal cycling under 200 MPa (Figure 6b–d). Furthermore, the nano-grained Ni–Ti–Cu SMAs reported by Dang et al. [71] also exhibited an improved cyclic stability with a transformation strain of ~4.8% and constant transformation temperatures (T1 and T2) during 50 cycles under 250 MPa (Figure 6e,f). The higher transformation strain levels of nano-grained ternary NiTi-based SMAs under constant tensile stresses are attributed to the local internal stresses induced by the favorable deformation substructures and refined grain size. The grain refinement and rearrangement of dislocation upon annealing result in the strengthening of the alloys, which leads to less plastic strain during thermal cycling under a constant load [35,71]. Moreover, the improvement in lattice compatibility brought by the addition of a third alloying element and the strengthening effect provided by the nano-grained structure enhanced the cyclic stability of the nanocrystalline ternary NiTi-based SMAs [37,71,72,73,74].

In the nanocrystalline ternary NiTi-based SMAs, it is concluded that introducing a third alloying element significantly improves the thermal stability of the phase transformation of nanocrystalline NiTi-based SMAs. The improved lattice compatibility and local internal stresses due to the addition of alloying elements reduce the generation of defects during phase transformation. Therefore, the nanocrystalline ternary NiTi-based SMAs reveal excellent reversibility and cycle stability during the thermally induced martensitic transformation.

## 3. Multicomponent Ultrafine NiTi-Based Shape Memory Alloys Fabricated Using an Amorphous Precursor

### 3.1. Basic Principle and Crystallization Mechanism of Amorphous Alloys

**Figure 7:**
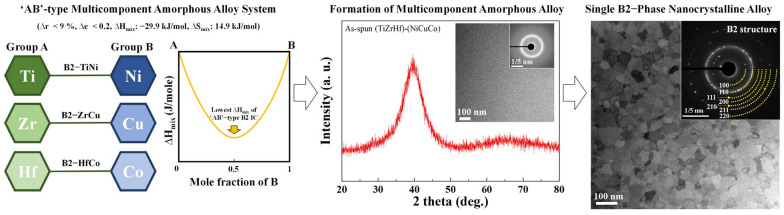
An example study to develop a nanocrystalline alloy using AAP with a polymorphous crystallization mechanism. Schematics for the alloy design strategy for the multicomponent amorphous alloy system and the structural characteristics of an ‘AB’-type multicomponent AAP with its crystallized single B2-phase nanocrystalline alloy [77].

AAs, referred to as metallic glasses, are a class of materials produced by the rapid quenching of molten alloys, which are considered to be a frozen solid in a liquid structure. Owing to their amorphous nature, AAs have interesting properties such as high strength, a large elastic strain limit, excellent wear and corrosion resistance, and biocompatibility [74,75,76]. Particularly, the meta-stable supercooled liquid state between T_g_ and T_x_ can be used as a precursor for the development of nanocrystalline or ultrafine-structured alloys by controlling its crystallization kinetics [38,39,77]. For example, Hong et al. [77] reported the development of nanocrystalline single-phase alloys using AAP with a multicomponent alloy system. To control the crystallization mechanism of the multicomponent alloy system, the (TiZrHf)–(NiCuCo) alloy system was designed as shown in Figure 7. The combination between ‘A’ and ‘B’ element groups can also be represented as ‘AB’-type combinations, such as TiNi, CuZr, and HfCo, which have the lowest Gibbs free energies and enthalpies in the liquid state as well as a B2 crystal structure. Moreover, the large negative mixing enthalpy (ΔH_mix_) and high mixing entropy (ΔS_mix_) between the ‘A’ and ‘B’ groups of the alloy are effective in stabilizing the liquid state at low temperatures to induce the formation of a monolithic amorphous structure. Moreover, the low atomic radius difference, almost zero enthalpy of mixing, similar electronegativity difference, and isomorphic crystal structure in each group consider Hume−Rothery rules for the formation of substitutional single-phase alloys. From the alloy design strategy considering physical and thermodynamic relations between constituent elements, a multicomponent AAP was fabricated successfully, and the AAP crystallized into a nanocrystalline single B2-phase alloy via a suitable annealing process (Figure 7). This result demonstrates the possibility of the development of multicomponent ultrafine NiTi-based SMAs using AAPs with a suitable crystallization mechanism and alloy design strategy.

To explore and advance the glass-forming ability (GFA) and the mechanical and physical properties of the AAs, numerous AAs have been discovered through compositional exploration with various developing methods [78,79,80], and a number of criteria to predict the glass-forming ability (GFA) have been proposed considering the thermodynamic parameters and atomic confusion, including the width of the supercooled liquid region (SCLR, ΔT*_x_*); a reduced glass transition temperature (T_rg_); the γ, β, σ, ω parameters that consider stability in the liquid phase and resistance to crystallization; the thermal stability of the amorphous phase; the related thermodynamic stability and atomic configuration of the liquid phase; the stability of the liquid and crystal and the competition of the amorphous and crystal, respectively; and so on [81,82,83,84,85,86]. Researchers have made great efforts to form bulk AAs with diameters on the millimeter scale, while Inoue [87] proposed empirical rules to design bulk AAs in a multicomponent alloy system.

Since the amorphous state is essentially metastable, it inherently possesses the possibility of transformation into a more stable crystalline state. However, the most excellent chemical and physical properties of AAs (mentioned above) have been found to deteriorate drastically during crystallization. Therefore, understanding the micro mechanisms of crystallization to impede or control crystallization is a prerequisite for designing partially or fully crystallized microstructures that cannot be obtained from liquid or crystalline states. The crystallization of AAs occurs generally through nucleation and growth processes. Due to the multicomponent alloy systems and compositional range of amorphously forming alloys that were designed to enhance the GFA and thermal stability, most AAs were crystallized through complicated crystallization reactions, i.e., the formation of a multi-phase structure via a decomposition reaction.

**Figure 8:**
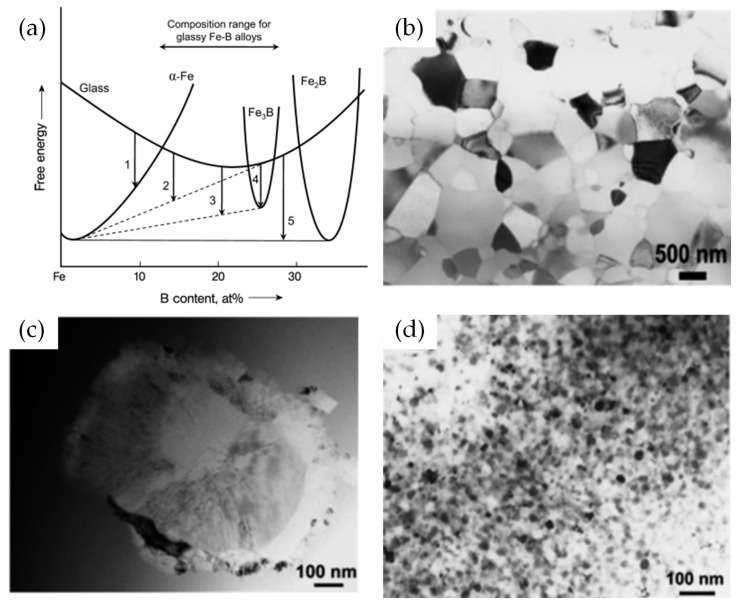
(**a**) Hypothetical free energy vs. composition diagram for the Fe-rich Fe–B alloy system, (where reactions 1 and 4 are polymorphous crystallization, reaction 2 is primary crystallization, and reactions 3 and 5 are eutectic crystallization, respectively). TEM BF images obtained after the (**b**) polymorphic, (**c**) eutectic, and (**d**) primary crystallization of AAs [88].

The hypothetical free energy vs. composition diagram [88] for the amorphous (glass) and different crystalline phases in the Fe-rich Fe–B system explain the reactions that occur during the crystallization of the AAs (Figure 8a). In this diagram, the variation in free energy with composition is presented for the glass, the α-Fe, the equilibrium Fe_2_B, and the frequently detected metastable Fe_3_B phases. Depending on the composition of the AAs, the crystallization into the stable equilibrium phases follows one of three reactions: (1) a polymorphous reaction; (2) a eutectic reaction; (3) a primary reaction. The typical microstructures of alloys crystallized from AAs through the three crystallization mechanisms are shown in Figure 8b–d. In the polymorphous crystallization, the amorphous phase transforms into a single crystalline phase without any change in composition (Figure 8b). The polymorphous crystallization occurs only in composition ranges where the amorphous phase has formed with a composition corresponding to a solid-solution phase or intermetallic compound. During polymorphous crystallization, the growth of crystals is linear with time and the growth rate has an Arrhenius dependence on temperature [89,90]. The eutectic crystallization of the amorphous phase is accompanied by transformation into two or more crystalline phases through a discontinuous reaction (Figure 8c), which can occur in the composition range between the two stable or metastable phases. For this reaction, the growth rate is independent of time until a hard impingement with another crystal occurs. For the primary crystallization of AAs, a supersaturated solid solution forms first from the amorphous phase (Figure 8d). Since the solute concentration is lower in the solid solution phase than that in the amorphous phase, the solute atoms are rejected in the amorphous phase, and consequently, the remaining glassy phase becomes enriched in the solute element until further crystallization is stopped. The solute-enriched amorphous phase transforms later through one of the crystallization mechanisms described above.

Understanding the crystallization mechanisms of AAs described above, it was concluded that the crystallization mechanism of the AAs is a selectable factor which is determined by alloy composition. The crystallization kinetics, such as nucleation and growth rates, can be controlled by modulating the annealing condition in the supercooled liquid state [91,92,93]. In recent decades, most amorphous alloy systems have been developed based on eutectic compositions to stabilize the liquid phase against lower temperatures and contain minor elements such as Al, Si, B, Sn, etc., that have large negative mixing enthalpy with major alloying elements to improve GFA [75,76,78,79,80]. The addition of minor elements results in the complexation of the alloy composition and induces the formation of brittle intermetallic compounds after crystallization [94,95,96]. To develop nanocrystalline or ultrafine NiTi-based SMAs through the crystallization of AAPs, it is necessary to design amorphous alloy systems with a polymorphous crystallization mechanism and suitable GFA.

### 3.2. Multicomponent Ultrafine NiTi-Based SMAs Synthesized Using Amorphous Thin Film

To fabricate thin NiTi films, various physical vapor deposition (PVD) techniques have been used [97,98,99,100]. The most commonly used PVD process is the magnetron sputtering technique, since it enables deposition at a rate of approximately 10 μm/h combined with relatively simple process equipment.

Since the as-deposited NiTi thin films are in a commonly amorphous state and require annealing processing to transform into their crystalline SMAs, the crystallization behavior of NiTi thin films has been considered an important scientific concern. The crystallization temperature depends on the composition of the as-deposited NiTi thin film, and the grain size of crystallized films is usually less than ~4 μm, primarily 1 μm, depending on the annealing conditions for crystallization [101]. The NiTi SMA thin film crystallized from the amorphous state exhibits obvious phase transformation behavior during thermal cycling similar to the bulk SMAs [100,102,103,104]. Since the microstructure, transformation behaviors, and mechanical properties of the NiTi SMA thin film are essentially the same as those in bulk materials, transformation and shape memory characteristics were investigated.

Many studies have investigated the behavior of NiTi SMA thin films for their prospective use in micro-electro-mechanical systems (MEMS) owing to their ability to recover large transformation stress and strain during thermal cycling, as well as their high actuation rate and work output-to-volume ratio compared to other types of actuators [105]. Although considerable breakthroughs have been achieved through extensive research, there are still some concerns regarding the use of these films in industrial applications on account of the inability to precisely control their response to external stimuli. Moreover, in addition to this limitation, low hardness and wear resistance further limit their field of application. To counter these deficiencies, much effort has been directed to develop more attractive NiTi SMA thin films, and the use of a third element has emerged as an effective approach to improving their properties.

**Figure 9:**
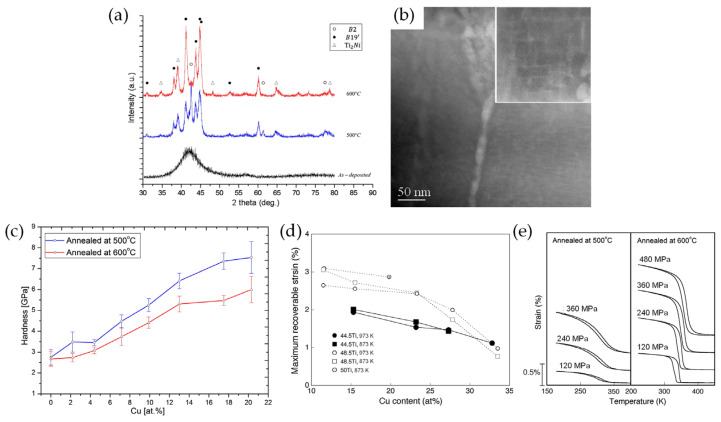
(**a**) XRD patterns of as-deposited and annealed Ni–Ti–Cu thin films, (**b**) TEM image, (**c**) hardness change as a function of Cu concentration, (**d**) maximum recoverable strain as a function of Cu concentration, (**e**) strain–temperature curves of annealed Ni–Ti–Cu SMA thin films [106,107].

Among the possible candidate alloying elements, Cu is one of the most promising as it reduces the compositional sensitivity of the transformation temperatures, reduces the temperature hysteresis, stabilizes the shape memory effect, and increases the actuation rate and the recoverable strain (mentioned in Section 2). For the Ni–Ti–Cu thin films, the as-deposited films formed an amorphous structure (as shown in the XRD patterns in Figure 9a). After annealing at 500 °C and 600 °C for 1 h, the amorphous thin films crystallized into a multi-phase structure consisting of B2 austenitic, B19’ martensitic, and a small number of Ti_2_Ni phases (Figure 9a). The martensite formed in the austenite matrix during cooling from the annealing temperature. This coexistence of both martensite and austenite is due to incomplete martensitic transformation at the ambient temperature, i.e., the martensite finish temperature (M_f_) is lower than the ambient temperature. The phase transformation temperatures of NiTi-based SMAs strongly depend on the chemical composition. The annealed thin film at 600 °C is composed of a much higher volume fraction of martensite and Ti_2_Ni phases than the thin film annealed at 500 °C, due to an increase in M_f_ due to the formation of Ti-rich precipitates in the matrix inducing a change in the chemical composition of the matrix. In addition, the nanoscale lath Ti_2_Ni phase was densely precipitated in the matrix (Figure 9b), which significantly improved the mechanical properties of the Ni–Ti–Cu thin films. The increase in the volume fraction of Ti_2_Ni precipitate with increasing Cu content improved the hardness of the SMA thin films, whereas the increasing amount of martensite degraded the precipitation-hardening effect (Figure 9c) [106]. The shape memory behavior of Ni–Ti–Cu SMA thin films is also influenced by the Cu content and annealing temperature. The maximum recoverable strains of the films decreased with increasing Cu content because of a decrease in the lattice deformation (Figure 9d) [107,108]. In addition, the martensitic transformation from the B19 phase to the B19’ phase overlapped with that from the B2 phase to the B19 phase for the Ni–Ti–Cu SMA thin film annealed at 600 °C with a high Cu content [109], which was attributed to the additional recoverable strain (Figure 9e).

**Figure 10:**
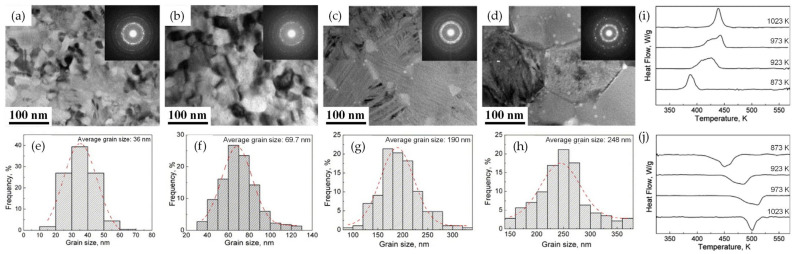
(**a**–**d**) TEM BF images with an inset selected-area electron diffraction (SAED) pattern, (**e**–**h**) histograms of grain size distributions, DSC cooling (**i**) and heating (**j**) curves of the nanocrystalline Ni–Ti–Hf thin films annealed at 600, 650, 700, 750 °C for 25 s [111].

The influence of the alloying element and annealing conditions on the phase transformation behavior and crystallization behavior of NiTi-based SMA thin films was further studied in Ni–Ti–Hf thin films [110,111,112]. The crystallization temperatures and activation energy for crystallization increased with the increasing Hf content due to an increase in the thermal stability of the amorphous thin film due to the addition of Hf, which caused larger atomic size mismatch and stronger interaction among the constituent elements [110]. This indicated that the addition of Hf improved the thermal stability of NiTi-based amorphous thin films, which is contrary to the role of Cu addition in the Ni–Ti–Cu thin film. For the investigation of different annealing conditions, the T_x_ and crystallization peak temperature (T_p_) shifted to higher temperatures with an increasing heating rate, implying that the crystallization process is dependent on the heating rate. During isothermal annealing, the grain size of crystallized Ni–Ti–Hf thin films significantly depended on the isothermal annealing temperature. The average grain increased from 36 nm to 248 nm when the annealing temperature increased from 600 °C to 750 °C (Figure 10a,d), which is a much smaller increase than that of the Ni–Ti–Cu thin film annealed at 500 °C and 600 °C. The variation in the grain size of Ni–Ti–Hf thin films, which strongly depends on the annealing temperature, also significantly influences thermally induced phase transformation behavior in the thin films. Due to the large number of defects such as dislocation, the grain boundary in nanocrystalline alloys increases the nucleation barrier. Therefore, the decrease in grain size in nanocrystalline Ni–Ti–Hf thin films resulted in decreases in the transformation temperature similar to the bulk NiTi SMAs [35,71,111,112].

### 3.3. Formation and Characterization of Ultrafine NiTi-Based SMAs Using Amrophous Alloy Ribbons as Precursors

Melt-spinning is a kind of rapid quenching method for casting AAs and an easy way to prepare NiTi-based alloys with sheet or ribbon forms. From the many studies on the development of NiTi-based SMAs using the thin film process, numerous NiTi-based alloy compositions to form amorphous structures have been discovered. The melt-spinning process can provide an easier way to control the alloy composition, with a shorter processing time and lower cost. However, it is difficult to obtain the amorphous structure in the binary NiTi alloy composition through the melt-spinning process due to the low GFA. Therefore, ternary or the higher multicomponent amorphous-forming alloy systems with high GFA are necessary to fabricate ultrafine NiTi-based SMAs using AAP. Among the ternary NiTi-based SMAs fabricated through the melt-spinning process, only the Ni–Ti–Cu alloys successfully fabricated the AAPs with monolithic amorphous structures [113]. Particularly, the Ni_25_Ti_50_Cu_25_ SMA fabricated using melt-spun AAP exhibited excellent shape memory properties, such as low compositional sensitivity of its transformation temperatures and small transformation hysteresis.

In accordance with the empirical rules for the formation of bulk AAs [87], the GFA of the AAPs for SMA can be increased through multicomponent alloying. However, the melt-spun multicomponent Ni–Ti–Zr [114], Ni–Ti–Hf–Cu, and Ni–Ti–Hf–Cu–Zr [115] AAPs usually exhibited primary crystallization behavior during annealing, i.e., the APPs crystallized into dual- or multi-phase structures, including B2, B19’, and unexpected intermetallic compounds. Therefore, the composition range of AAPs should be strictly limited to secure their polymorphous crystallization into a single-phase structure such as NiTi.

From the great effort to develop AAPs with good GFA and thermal stability, AAPs with a polymorphic crystallization mechanism were found from the Ni–Ti–Hf–Cu and Ni–Ti–Cu–Zr alloy systems. Ochin et al. [116] reported that the increase in the degree of dense random packed structure is reflected in the difference in the atomic size distribution plots (ASDP) for the “ordinary” amorphous phase. For a multielementary pseudo-binary AB alloy with appropriate ASPD, the largest GFA range should fit the requirements for SMA. The multicomponent pseudo-binary AB-type intermetallic compound system was designed by selecting possible alloying elements (“A” = Ti, Zr, Hf; “B” = Ni, Co, Cu, Pd, Sn). By substituting Hf instead of Ti alongside the substitution of Cu for Ni, melt-spun Ti_32_Hf_18_Ni_35_Cu_15_ AAPs with high GFA were fabricated, which crystallized polymorphically into a single-phase SMA with a B2 structure and exhibited obvious SE [116].

**Figure 11:**
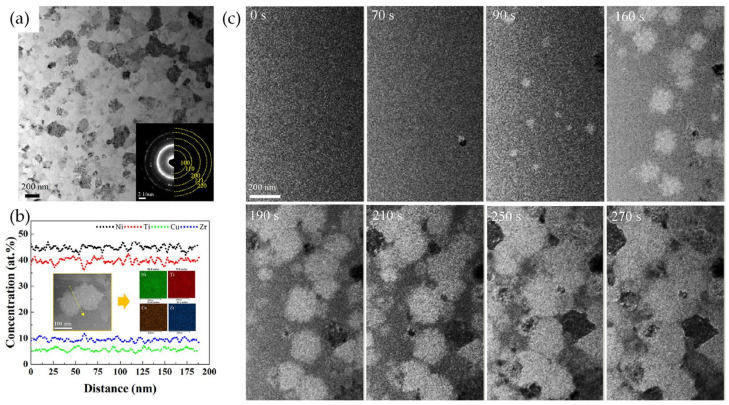
(**a**) TEM BF image with an inset SAED pattern, (**b**) EDS line profiles with inset elemental distribution maps of crystallized Ni_45_Ti_40_Cu_5_Zr_10_ SMA, (**c**) TEM BF images obtained during isothermal annealing using in situ TEM experiments on melt-spun Ni_45_Ti_40_Cu_5_Zr_10_ AAP [93].

The influence of the chemical composition on GFA and the crystallization mechanism of melt-spun AAPs was studied on the above-mentioned alloys. To obtain deep insight into the relationship between the annealing condition and crystallization kinetics of AAPs, the crystallization behavior of the melt-spun AAPs was systematically investigated in the melt-spun Ni–Ti–Cu–Zr AAPs with polymorphous crystallization reactions [93]. Since the Ni–Cu and Ti–Zr binary systems with complete solid soluble relationships are in a state of very similar electronegativity difference and have a low atomic radius difference, respectively, the Cu and Zr were suggested as substitutional solutes for a multicomponent NiTi-based alloy system. Additionally, it was expected that the large negative mixing enthalpy between the constitutive elements that satisfies Inoue’s empirical rules to design AAs [89] is effective to improve the GFA of NiTi-based alloy systems. The melt-spun Ni_45_Ti_40_Cu_5_Zr_10_ alloy ribbon with monolithic amorphous structure crystallized into single B2-phase SMAs with homogeneous chemical distributions (Figure 11a,b) via polymorphous reactions, which were systematically investigated through in situ TEM experiments (Figure 11c) [93].

**Figure 12:**
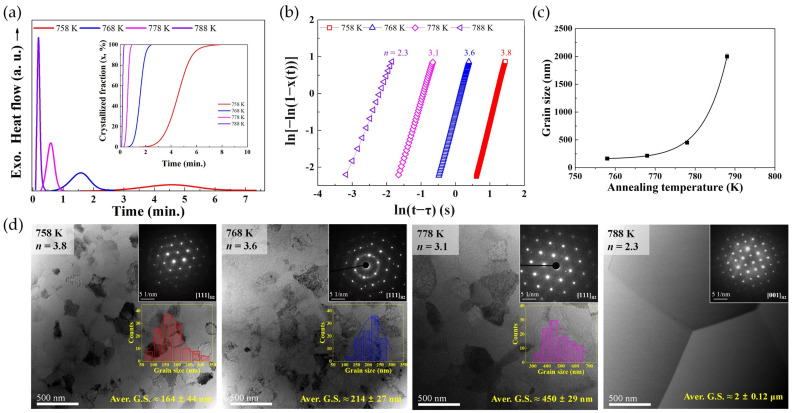
(**a**) Isothermal DSC curves obtained from different annealing temperatures, with inset crystallization fraction curves as a function of time, (**b**) Avrami exponent (*n*) value depending on the annealing temperature, (**c**) temperature dependence of grain size, (**d**) TEM BF images of crystallized SMAs produced through isothermal annealing of Ni–Ti–Cu–Zr AAPs [93].

The melt-spun Ni_45_Ti_40_Cu_5_Zr_10_ AAP exhibited the large temperature range of SCLR, which provided an opportunity to investigate the crystallization kinetics across a wide temperature range. From the systematic isothermal annealing experiment in a temperature range from 758 K to 788 K (Figure 12a), the crystallization kinetics of the AAP were understood through evaluation of the Avrami exponent (*n*) of the Johson−Mehl−Avrami theory [117] (Figure 12b), which demonstrated that melt-spun Ni_45_Ti_40_Cu_5_Zr_10_ AAP was crystallized by interface-controlled three-dimensional growth with a decreasing nucleation rate at a temperature ranging between 758 K and 778 K, whereas crystallization kinetics at higher annealing temperature turned to diffusion-controlled three-dimensional growth with a decreasing nucleation rate. In addition, the annealing temperature dependence of the grain size melt-spun Ni_45_Ti_40_Cu_5_Zr_10_ AAP was also demonstrated through the classical nucleation theory and temperature dependence of the growth rate. The lower isothermal annealing temperature induced a higher nucleation rate by decreasing the free energy barrier of nucleation with a lower growth rate. Therefore, the average grain size of the B2 austenite phase in the crystallized Ni_45_Ti_40_Cu_5_Zr_10_ SMAs exponentially decreased from 2 μm to 164 nm with the decreasing annealing temperature (Figure 12c,d). Based on the above systematic investigations of Ni_45_Ti_40_Cu_5_Zr_10_ AAP, Hong et al. [93] concluded that the crystallization kinetics of multicomponent Ni–Ti–Cu–Zr AAPs are strongly influenced by the isothermal annealing temperature due to the significant temperature dependence of nucleation and growth rates.

**Figure 13:**
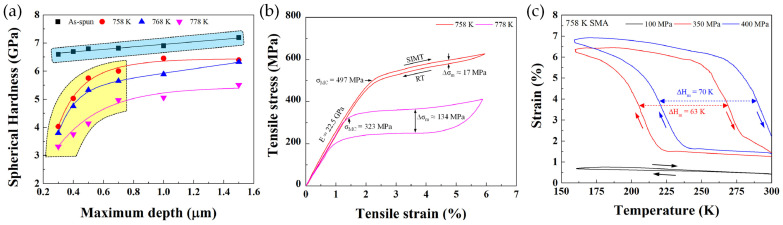
(**a**) Spherical nanoindentation hardness–depth curves of AAP and ultrafine Ni–Ti–Cu–Zr SMAs, where the hardness change in the yellow area is a response of stress-induced martensitic transformation, (**b**) stress−strain response during tensile stress cyclic loading, (**c**) cyclic strain–temperature curves under different tensile stress levels in ultrafine Ni–Ti–Cu–Zr SMAs [93].

For the thermomechanical responses, the ultrafine Ni_45_Ti_40_Cu_5_Zr_10_ SMAs fabricated through the crystallization of their AAPs exhibited excellent strain recovery behaviors such as (1) martensitic phase transformation behavior during indentation loading (Figure 13a), (2) a completely recoverable strain of ~6% during tensile stress cyclic loading (Figure 13b), and (3) a large recovery strain during thermal cycling under static tensile stress conditions (Figure 13c). In addition, the mechanical properties and strain recovery behavior of the SMAs strongly depended on their grain size. Therefore, the annealing temperature for the crystallization of AAPs is considered a very important factor to tailor the grain size and thermomechanical properties of the ultrafine SMAs fabricated using AAPs.

**Figure 14:**
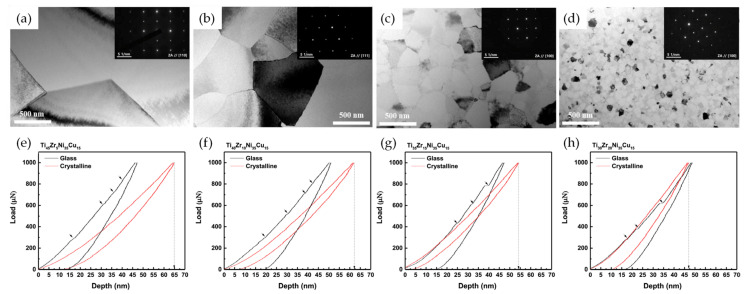
(**a**–**d**) TEM BF images of crystallized Ni_35_Ti_50-x_Zr_x_Cu_15_ SMAs (x = 5, 10, 15, 20 at.%), (**e**–**h**) load−depth curves obtained from nanoindentation of the melt-spun AAPs and their crystallized SMAs, where the arrows indicate pop-in events corresponding to the propagation of shear bands [118].

In the AAPs for SMA, the alloy composition is also important to determine the crystallization kinetics. Kim et al. [118] systematically demonstrated the effect of alloying elements on the crystallization kinetics in the melt-spun Ni–Ti–Zr–Cu AAPs through substitution of Zr for Ti. With regard to the crystallization kinetics of melt-spun Ni_35_Ti_50-x_Zr_x_Cu_15_ AAPs with x = 5, 10, 15, 20 at.%, Zr acts as an element that hinders crystal growth due to its large atomic radius and low diffusivity. The increase in Zr content increases the GFA of the AAPs. The average *n* value of the AAPs that classifies the crystallization kinetics such as the degree of nucleation rate and crystal growth mode [117] decreased with increasing Zr content from 3.18 to 3.17, which reflected that Ni_35_Ti_50-x_Zr_x_Cu_15_ AAPs were crystallized through interface-controlled three-dimensional growth with a decreasing nucleation rate. This trend was also reflected in the decrease in the grain size of the crystallized Ni_35_Ti_50-x_Zr_x_Cu_15_ SMAs via an isothermal annealing process; the grain size drastically decreased from 2.6 μm to 44.9 nm (Figure 14a–d). Moreover, the increase in Zr content induced the formation of a wavy grain boundary. These microstructural evolutions, such as grain refinement and wavy grain boundaries, could suppress the martensitic transformation by changing the thermodynamic balance condition [118,119]. For the strain recovery behavior of SMAs, the grain refinement of SMAs brings a positive effect due to the enhanced resistance to slip deformation. Therefore, the superelastic response of Ni_35_Ti_50-x_Zr_x_Cu_15_ SMAs was improved due to the decrease in the grain size to the nanoscale with increasing Zr content, as shown in the mechanical response to nanoindentation (Figure 14e–h).

**Figure 15:**
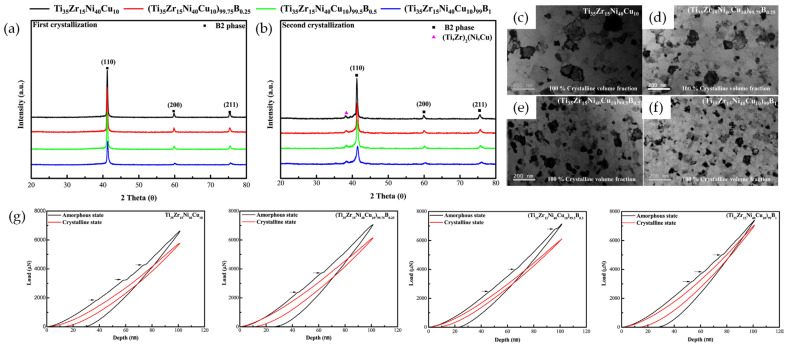
XRD patterns of (**a**) the first and (**b**) second crystallized (Ni_40_Ti_35_Zr_15_Cu_10_)_100−x_B_x_ SMAs (x = 5, 10, 15, 20 at.%), (**c**–**f**) TEM BF images of completely crystallized SMAs, (**g**) load–depth curves obtained through the nanoindentation of melt-spun AAPs and crystallized SMAs, where the arrows indicate pop-in events corresponding to the propagation of shear bands [120].

Recently, the effect of minor alloying on GFA, crystallization behavior, and superelastic properties has been reported in the Ni–Ti–Zr–Cu–B alloy system [120]. The small addition of B content in (Ni_40_Ti_35_Zr_15_Cu_10_)_100−x_B_x_ (x = 0, 0.25, 0.5, 1 at.%) AAPs increased GFA due to the addition of B with a small atomic radius and large negative mixing enthalpy. Moreover, The SCLR of AAPs was stabilized by an increase in the T_x_ and activation energy for phase transformation. The crystallization kinetics of the AAPs is drastically influenced by the addition of B content. The crystallization mechanism was changed from polymorphous to dendritic crystallization with a compositional change through solute partitioning during crystallization. According to the change in crystallization behavior, Ni–Ti–Zr–Cu–B AAPs undergo two-step crystallization. The B2 phase formed during the first crystallization and the (Ti,Zr)_2_(Ni,Cu) intermetallic compound formed a residual amorphous phase during the second crystallization (Figure 15a,b). The initial nucleation rate was accelerated while crystal growth was hindered. Thus, the average grain size of the crystallized SMAs decreased from 250 nm to 70 nm as B content increased up to 1 at.% (Figure 15c–f). For the mechanical response of the crystallized SMAs, the nanocrystalline SMAs exhibited SE during nanoindentation, and the SMAs with 0.5 at.% B showed the highest SE at room temperature (Figure 15g).

To fabricate the millimeter-scale multicomponent ultrafine NiTi-based SMAs from the AAPs, additional forming processes, such as hot pressing (HP) and spark plasma sintering (SPS), have been suggested. Kim et al. [121] reported that for Ni–Ti–Cu–Zr–Si AAP. the addition of Si content up to 2 at.% in Ni_35_Ti_30_Cu_15_Zr_20_ alloy improved the GFA up to 450 μm and effectively stabilized the SCL with a significant increase in the activation energy for the glass transition, which increased the incubation time at T_g_ before crystallization. The improved incubation time at T_g_ facilitated the thermoplastic-forming process of the Ni–Ti–Cu–Zr–Si AAPs. By hot pressing the overlapped as-cast amorphous plates, an AAP with a millimeter-scale thickness could be achieved. Cai et al. [122] reported the development of an ultrafine Ni–Ti–Zr–Cu SMA using the SPS method on an AAP with a thickness of 70 μm. The grain size of the as-sintered bulk SMAs increased from 250 nm to 450 nm with an increase in the sintering temperature from 723 K to 923 K. The as-sintered bulk SMA with ultrafine grains exhibited perfect superelasticity with a high recoverable strain of ~5.8%, which is attributed to the high resistance to grain boundary slip or dislocation slip generated in the austenite matrix from the secondary phase of the residual nanoscale amorphous phase.

In the case of SMAs fabricated through crystallization of AAPs at relatively low temperature, i.e., SCLR, the crystallization kinetics of AAPs is an important factor in determining the microstructural features such as phase formation, grain size, and grain boundary morphology, which are closely related to the SME, SE, and transformation behavior. Since the crystallization kinetics are highly dependent on the annealing temperature and alloy composition, systematic investigations concerning optimizing the annealing condition and compositional tuning were undertaken to optimize the overall strain recovery behavior. From intensive studies on multicomponent NiTi-based AAPs, ultrafine SMAs with grain sizes ranging from few nanometers to few micrometers have developed. The lower annealing temperature induces a higher nucleation rate with a lower growth rate during crystallization, which results in the formation of nano-grained multicomponent NiTi-based SMAs. Additionally, composition tuning of Ni–Ti–Cu–Zr AAPs and minor alloying are effective to enhance the GFA of AAs and the formation of much smaller grains by hindering crystal growth during crystallization. The nanocrystalline structure of multicomponent NiTi-based SMAs drastically improves the shape memory and superelastic behaviors with high strength.

## 4. Conclusions

NiTi SMAs, considered to be one of the most representative smart materials, have been used in a broad range of industrial engineering applications due to their unique SME and SE. Due to the requirements of high-tech industries, SMAs must be advanced, with larger recovery strains, higher cycling stability and transformation temperatures, improved workability, and enhanced micro/nanostructure fabrication characteristics. The physical properties of NiTi-based SMAs have been improved and controlled through multicomponent alloying methods and fabrication process development. In particular, multicomponent alloying in SMAs has been introduced since not only the shape memory properties can be enhanced by minor alloying addition and interatomic interactions, but the glassy precursor for SMAs can be fabricated. From this perspective, we discussed recent advances in the development of ultrafine or nanocrystalline NiTi-based SMAs fabricated using the thermomechanical process and crystallization process from the AAPs.

To reduce the grain size of NiTi-based SMAs to the nanoscale, thermomechanical processing was applied. Through SPD processing using NiTi SMAs with coarse grains, the grain size of the NiTi SMAs was refined to 10 nm. The grain size of as-rolled NiTi SMAs was tailored in the nanoscale (10–80 nm) through heat treatment accompanying the recovery of the deformed lattice and recrystallization. Therefore, the nanocrystalline SMAs have a highly dense grain boundary, which leads to grain refinement strengthening and improved cyclic and dynamic stability. With decreasing grain size, the transformation strain decreases due to a gradual suppression of stress-induced phase transformation by increasing phase transformation stress. The grain size reduction below critical size stabilizes the alloys by introducing specific interfacial energy. Therefore, the *H* tends to vanish. Moreover, the fatigue life of superelastic SMAs is significantly improved with a grain size reduction, which is mainly attributed to the strengthening and *H* reduction through grain size reduction. In addition, high resistance against the dislocation slip of nanocrystalline SMAs retarded the crack nucleation via the grain boundary-mediated mechanism. For the below-critical grain size of 10 nm, the transformation is completely hindered because the mechanical constraints imposed by neighboring grains with different orientations hinder nucleation in grain boundaries.

In the nanocrystalline ternary NiTi-based SMAs processed using the thermomechanical method, the alloying elements significantly improve the thermal stability of the phase transformation of nanocrystalline Ni–Ti–Hf and Ni–Ti–Cu SMAs. The improved lattice compatibility and local internal stresses due to the addition of alloying elements reduce the generation of defects during phase transformation. Through the effect of alloying elements on the thermal stability of phase transformation, the nanocrystalline Ni–Ti–Cu SMA reveals excellent reversibility and cyclic stability in the thermally induced martensitic transformation, which improves the SME.

Novel strategies to develop multicomponent ultrafine NiTi-based SMAs have been suggested, using the crystallization mechanism of AAs. The AAs usually crystallize into a nanocrystalline structure. Multicomponent NiTi-based AAs have been developed through systematic alloy design strategies to achieve polymorphous crystallization. For the Ni–Ti–Cu and Ni–Ti–Hf amorphous thin films, the AA films usually crystallize into a multi-phase structure composed of B2, B19’, and Ti_2_Ni phases. In the nanocrystalline Ni–Ti–Cu SMA thin film, the maximum recoverable strain decreases with the increase in Cu content due to a decrease in the lattice deformation. However, the overlapping of the two-step martensitic transformation is attributed to the improvement in recoverable strain.

Another way to fabricate AAPs is the melt-spinning method. The multicomponent pseudo-binary ‘AB’-type alloy system, similar to NiTi, improves the GFA and thermal stability of AAPs. From the systematic alloy design of the Ni–Ti–Cu–Zr alloy system, the melt-spun AAPs with a polymorphous crystallization mechanism crystallize into a single B2-phase structure. For the isothermal annealing between T_g_ and T_x_, the crystallization mechanism and nucleation and growth rates significantly depend on the annealing temperature. As a result, the grain size exponentially decreases with the decreasing annealing temperature, and nanocrystalline SMAs with a grain size of 164 nm are successfully obtained. Moreover, the nucleation and growth kinetics of the AAPs are significantly influenced by the concentrations of the constituents. An increasing Zr content hinders the crystal growth, which results in the formation of single B2-phase SMAs with a grain size of 44.9 nm. The nanocrystalline Ni–Ti–Cu–Zr SMAs exhibited a complete strain recovery of ~6% through reversible martensitic transformation during tensile cycling and thermal cycling under static loading. Based on this review, it is believed that the novel strategy to develop multicomponent ultrafine NiTi-based SMAs using AAPs can be considered an attractive simple processing method for engineering fields. Therefore, further intensive studies to improve the GFA of AAPs and to optimize the annealing condition for crystallization will facilitate the use of the ultrafine NiTi-based SMAs for diverse engineering applications as smart materials.

## Data Availability

No new data were created.
